# Three-Dimensional Mapping of Soil Organic Carbon by Combining Kriging Method with Profile Depth Function

**DOI:** 10.1371/journal.pone.0129038

**Published:** 2015-06-05

**Authors:** Chong Chen, Kelin Hu, Hong Li, Anping Yun, Baoguo Li

**Affiliations:** 1 Department of Soil and Water Sciences, China Agricultural University, No 2, Yuan Ming Yuan Xi Lu, Beijing 100193, China; 2 Environment and Plant Protection Institute, Chinese Academy of Tropical Agricultural Science, Haikou 571101, China; 3 Department of Plant and Animal Sciences, Nova Scotia Agricultural College, Truro, Nova Scotia, B2N 5E3 Canada; Chinese Academy of Sciences, CHINA

## Abstract

Understanding spatial variation of soil organic carbon (SOC) in three-dimensional direction is helpful for land use management. Due to the effect of profile depths and soil texture on vertical distribution of SOC, the stationary assumption for SOC cannot be met in the vertical direction. Therefore the three-dimensional (3D) ordinary kriging technique cannot be directly used to map the distribution of SOC at a regional scale. The objectives of this study were to map the 3D distribution of SOC at a regional scale by combining kriging method with the profile depth function of SOC (KPDF), and to explore the effects of soil texture and land use type on vertical distribution of SOC in a fluvial plain. A total of 605 samples were collected from 121 soil profiles (0.0 to 1.0 m, 0.20 m increment) in Quzhou County, China and SOC contents were determined for each soil sample. The KPDF method was used to obtain the 3D map of SOC at the county scale. The results showed that the exponential equation well described the vertical distribution of mean values of the SOC contents. The coefficients of determination, root mean squared error and mean prediction error between the measured and the predicted SOC contents were 0.52, 1.82 and -0.24 g kg^-1^ respectively, suggesting that the KPDF method could be used to produce a 3D map of SOC content. The surface SOC contents were high in the mid-west and south regions, and low values lay in the southeast corner. The SOC contents showed significant positive correlations between the five different depths and the correlations of SOC contents were larger in adjacent layers than in non-adjacent layers. Soil texture and land use type had significant effects on the spatial distribution of SOC. The influence of land use type was more important than that of soil texture in the surface soil, and soil texture played a more important role in influencing the SOC levels for 0.2-0.4 m layer.

## Introduction

Soil organic carbon (SOC) stock is an important carbon pool in terrestrial ecosystems and a main source of greenhouse gas. Soil organic carbon is closely related to soil structure and fertility and is commonly used as a key indicator for assessing soil quality [1, 2]. Therefore, quantitative evaluation of SOC levels is meaningful for sustainable soil utilization and management from estimating SOC stocks. Recent studies have been focused on the lateral (two-dimensional) distribution of SOC for estimate SOC stock in some regions [3–6]. As SOC content is also closely related to soil water retention characteristics and soil sorption capacity of pollutants [7, 8], understanding SOC spatial variation in three-dimensional (3D) direction would be helpful for assessing transport of nutrients and pollutants.

The variability of soil properties in 3D direction has been reported in several recent studies [9–21]. These studies provided some useful information about spatial variability of different soil properties in both horizontal and vertical directions, and the generated information can be used as model inputs [15]. Also, as many soils are developed from fluvial sediments, and the variability in soil texture and clay content in alluvial soils are complicated in both horizontal and vertical directions [11, 14], the distribution of SOC content may be significantly different in three dimensions. In addition, land use types had significant effects on spatial distribution of SOC [22–25].

Geostatistical tools can be directly used to map soil properties in 3D direction for many variables such as soil strength [9], soil nitrate concentration [10], soil texture [11], soil salinity [13] and soil clay content [14]. However, due to the effect of profile depths on vertical variation of SOC, the stationary assumption for SOC cannot be met in the vertical direction. Thus, geostatistical tools were seldom used for mapping SOC in three dimensions. There are many methods of combining lateral soil mapping with profile depth function fitting for acquiring the 3D information of SOC. The SOC stock (0–1 m) in the Lower Namoi Valley, New South Wales was mapped by fitting the exponential function to carbon profile data and predicting parameters of the function from environmental data using an artificial neural network technique [16]. The SOC stocks of three depth intervals (0–0.5 m, 0.5–1 m and 0–1 m) was mapped on a state scale by using negative exponential function to fit the vertical distribution of SOC and then utilizing ordinary kriging technique to interpolate the parameters of the functions [17]. Malone et al. [18] mapped the continuous depth functions of SOC storage in the lower valley of the Namoi River by combing the neural network model, kriging and the spline depth functions. Meersmans et al. [19] analyzed the 3D distribution of SOC density at the regional scale (15,521 km^2^). An exponential function was used to describe the vertical distribution of SOC and the parameters of the function were then calculated based on their empirical relationships with land uses and soil types. Liu et al. [20] mapped the 3D distribution of SOC in a subtropical hilly landscape (12 km^2^). The equal-area quadratic splines were used to fit the vertical distribution of SOC and the radial basis function (RBF) neural networks were used to predict the lateral distribution of SOC based on its relations with environmental variables. Lacoste et al. [21] mapped SOC content in three dimensions at the landscape scale (10 km^2^) by using a data mining tool (Cubist) to predict SOC content at eight fixed layers based on environmental covariates, and a spline function was then used to fit the vertical distribution of SOC content at each location.

However, above-mentioned methods had some disadvantages in 3D mapping because a large set of profile data needs to be separately fitted using a specific function and some profile data can not be fitted well using this function. There is a need to develop a suitable and efficient method to map the 3D spatial distribution of SOC content.

In this study, the Quzhou County with an area of 667 km^2^ in a fluvial plain was selected as our study area. We considered soil profile depths as a categorical information to remove its effects on the distribution of SOC, then mapped the 3D distribution of the residual using 3D ordinary kriging technique. Finally, the 3D distribution of SOC content were obtained by combined the residual with profile depth distribution function of SOC. The objectives of this study were (i) to provide a simple and reasonable method for mapping the 3D distribution of SOC, and (ii) to explore the effects of soil texture and land use type on spatial distribution of SOC in different depths.

## Materials and Methods

### Study area and data collection

The study was conducted in Quzhou County, south of Hebei Province, located between 36°35′43"- 36°57′56"N and 114°50′22″- 115°13′27″E. The study area is about 667 km^2^ and agricultural land accounts for over 80% of the area. The elevation ranges from 32.7 to 45.4 m within the area. The entire terrain is slightly inclined from southwest towards northeast. The county belongs to a continental monsoonal climate. The long-term annual mean air temperature is 13.1°C and the mean annual precipitation is 534.9 mm. The precipitation occurs mostly from June to September. The soils, formed principally from fluvial deposits, are classified as Fluventic Ustochrept in the USDA soil classification system [26]. In this region, the typical crop rotations are wheat–maize, wheat–cotton and wheat–soybean annually. Authors have the permission to conduct the study on this site, and confirm that the studies did not involve endangered or protected species.

**Fig 1:**
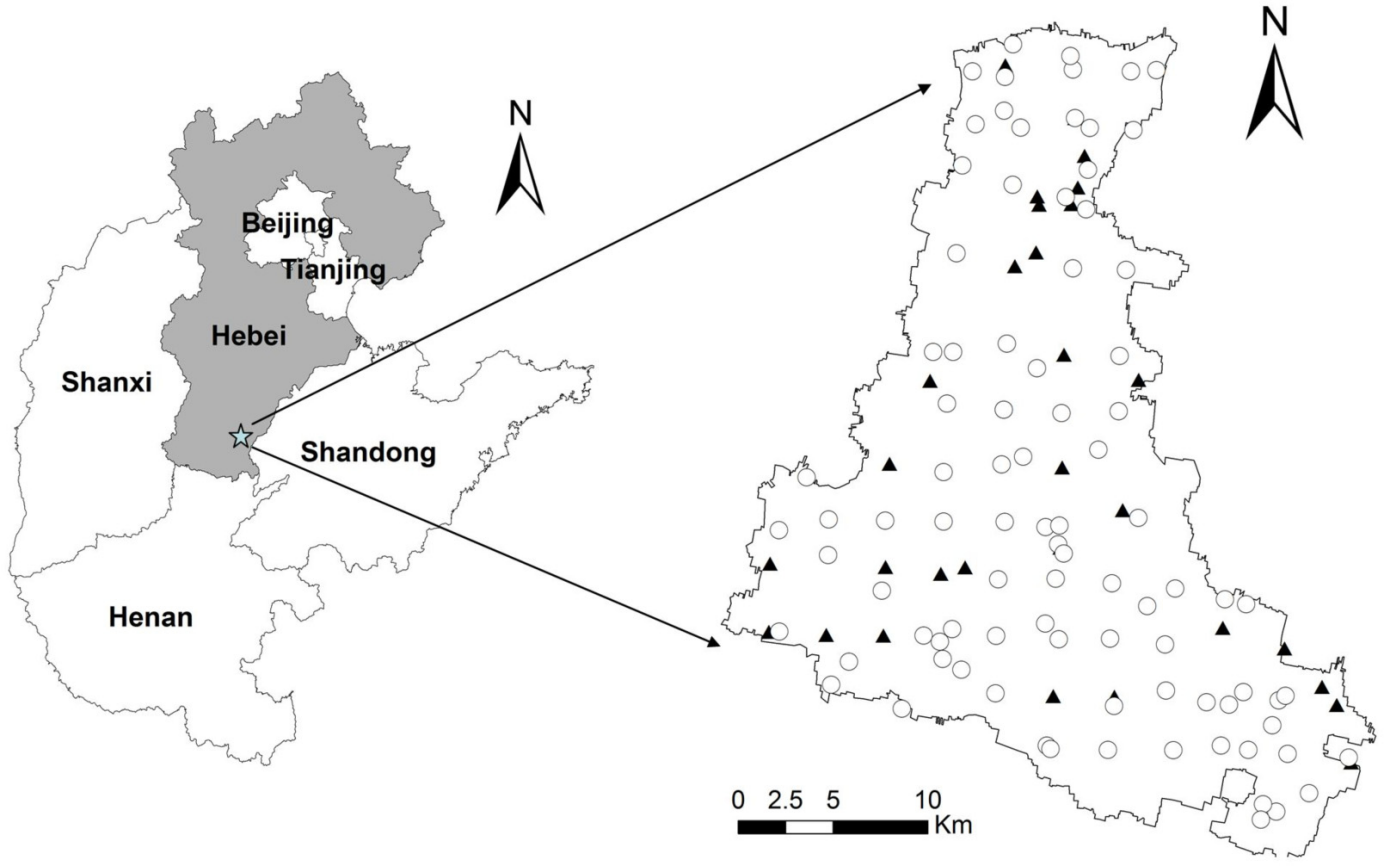
Locations of soil sampling sites in Quzhou County in the North China Plain (NCP). Open circles represent calibration data (91 profiles and 455 samples) and solid triangles represent validation sites (30 profiles and 150 samples).

A total of 121 soil profiles (0–1 m) were selected in the study region (Fig 1) by combining a grid sampling method with stratified sampling method (S1 Datasheet). We overlapped sampling grid (2.5 km×2.5 km) using the maps of soil classification and land use types. More specific soil sampling points were planned for the area with complex soil types and land use types and fewer soil sampling points were taken for flat areas. A portable global position system (GPS) was used to record soil sampling locations. At five depth intervals (0–0.2, 0.2–0.4, 0.4–0.6, 0.6–0.8 and 0.8–1 m), soils were sampled from the profiles using a soil auger in May 2010. Soil samples were air-dried, ground and passed through a 0.15 mm sieve for chemical analysis. Soil organic carbon content was determined using the dichromate oxidation method [27]. Based on the distribution maps of soil texture and land use types, a total of 150 samples from 30 soil profiles were taken from the whole set of data as validation dataset, the other 455 samples (91 profiles) were used as the calibration dataset (Fig 1).

### Three dimensional ordinary kriging

Three dimensional kriging requires calculating and fitting the three directional (*x*, *y*, *z*) experimental variograms. The directional variograms were modeled by considering data pairs only in one direction among the horizontal plane or vertical direction, and then one 3D model was formed by combining these directional variograms [10, 14, 28].

The kriging estimate of soil property is expressed as a linear weighted average of the *n* observations surrounding the location *x*:

$$Z^{*}(x)=\sum_{\alpha =1}^{n}\lambda_{\alpha }Z(x_{\alpha })$$ (1)

where *λ*_*α*_ represents the weight obtained by solving the ordinary kriging system [29].

### Three dimensional kriging combined with profile depth function (KPDF)

The data of SOC were usually collected from different profile depths. As shown in Liu et al. [30], the data of SOC can be divided into different classes (*d*_*j*_) based on the difference of profile depths. Thus, the profile depth information could be used to interpret the variation of SOC. For each observation *Z*(*x*_*αj*_), it can be expressed as the sum of the mean value of *Z*(*x*_*αj*_) at the profile depth *d*_*j*_ and its residual *r*(*x*_*αj*_):

$$Z(x_{\alpha j})=p(d_{j})+r(x_{\alpha j})$$ (2)

where *α* and *j* represent horizontal and vertical directional locations, respectively. *Z*(*x*_*αj*_) is SOC at the location of *x*_*αj*_, which is at the profile depth *d*_*j*_. *p*(*d*_*j*_) is the mean of *Z*(*x*_*αj*_) at the profile depth *d*_*j*_, and *r*(*x*_*αj*_) is the residual. *p*(*d*_*j*_) and *r*(*x*_*αj*_) are mutually independent, and the variation of *r*(*x*_*αj*_) is homogeneous at different profile depths. Thus, the variance of *Z*(*x*_*αj*_) can be written as:

$$\sigma_{z}^{2}=\sigma_{p}^{2}+\sigma_{r}^{2}$$ (3)

The variance *σ* ^2^_*p*_ between different profile depths indicates the effect of profile depths on the spatial variation of SOC. The variances *σ* ^2^_*r*_ shows the overall variation at different profile depths, which assumes homogeneous and satisfies the stationary assumption. Thus, the estimator Z*(*x*_*αj*_) can be summed as the kriging estimate of residual r*(*x*_*αj*_) and the estimate of profile depths p*(*d*_*j*_):

$$Z^{*}(x_{\alpha j})=p^{*}(d_{j})+r^{*}(x_{\alpha j})$$ (4)

**Fig 2:**
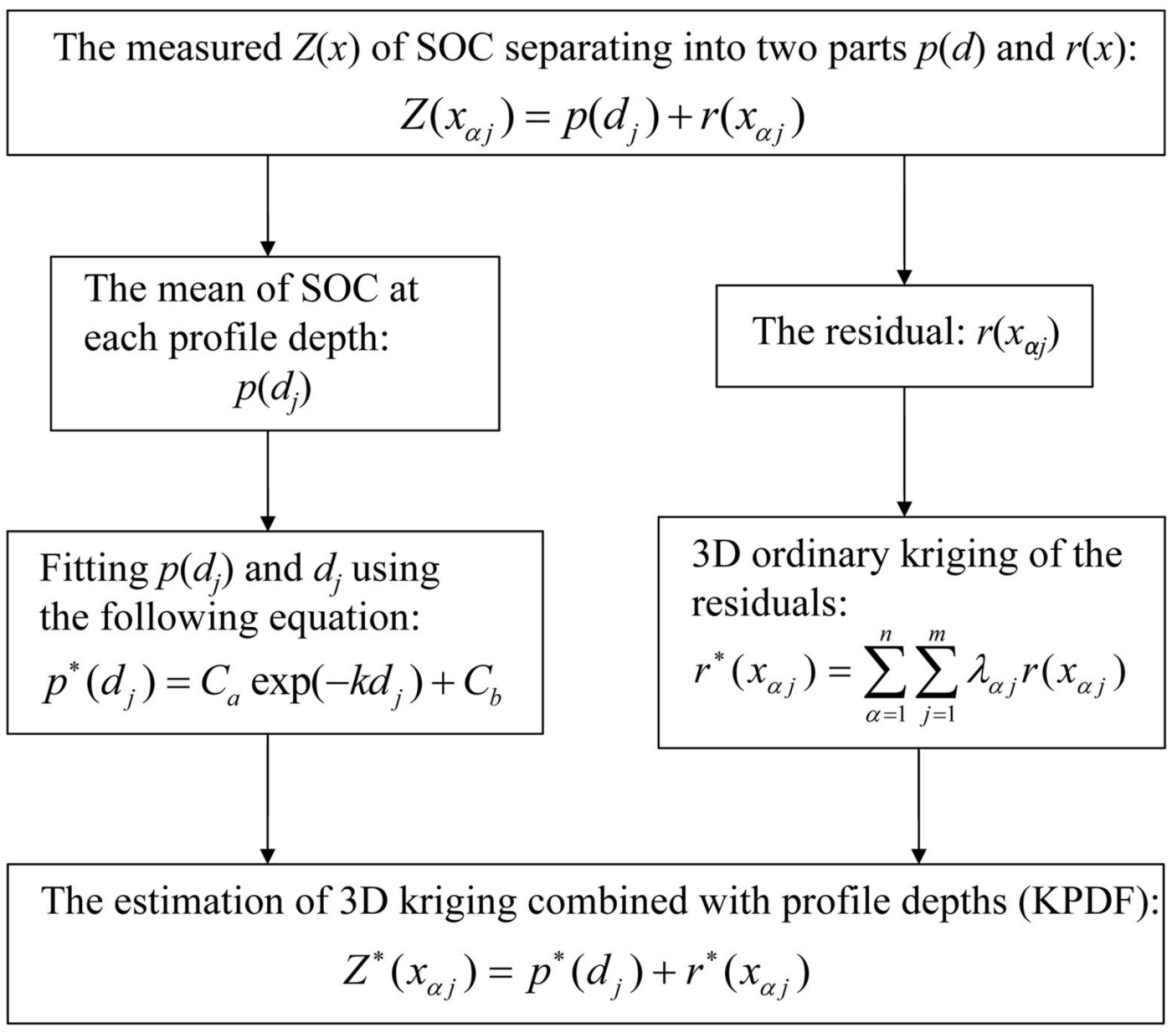
The flow chart of kriging combined with profile depth function of soil organic carbon (KPDF) interpolation procedures.

In this study, the mean value of SOC *p*(*d*_*j*_) at the profile depth *d*_*j*_ can be expressed as:

$$p(d_{j})=\frac{1}{m}\sum_{\alpha =1}^{m}Z(x_{\alpha j})$$ (5)

where *m* is the number of observed SOC at a depth of *d*_*j*_. Based on the mean value of SOC at the five known depths, we fit *p*(*d*_*j*_) and *d*_*j*_ using the following equation to get the mean value of SOC at any depth.

$$p^{*}(d_{j})=C_{a}\text{exp}(-kd_{j})+C_{b}$$ (6)

where *C*_*a*_, *C*_*b*_ and *k* are three fitting parameters. *k* determines the shape of the exponential part of the curve. The dominant land use types are farmland in the study area. Due to long-term tillage, the surface SOC is relative uniform by mixing. Thus, the mean value of SOC at a depth of less than 0.1m was set to the mean at a depth of 0.1m. The estimation of residual r*(*x*_*αj*_) is obtained by 3D ordinary kriging interpolation.

$$r^{*}(x_{\alpha j})=\sum_{j=1}^{n}\sum_{\alpha =1}^{m}\lambda_{\alpha j}r(x_{\alpha j})$$ (7)

where *λ*_*αj*_ is the weight obtained by solving the ordinary kriging system, *n* is the number of interpolation in vertical direction. The detailed interpolation steps are shown in Fig 2.

### Validation

Independent validation was used to evaluate the interpolation performance of 3D kriging combined with profile depth function. The validation results were assessed using common statistical indices, e.g. the coefficient of determination (*R* ^2^), the mean prediction error (ME) and the root mean square error (RMSE):

$$\text{ME}=\frac{1}{n}\sum_{i=1}^{n}\left(P_{i}-O_{i}\right)$$ (8)

$$\text{RMSE}=\sqrt{\sum_{i=1}^{n}\frac{{\left(P_{i}-O_{i}\right)}^{2}}{n}}$$ (9)

where *P*_*i*_ and *O*_*i*_ represent the predicted and observed SOC, respectively, and *n* is the number of measurements.

### Data process

The normality of the SOC datasets was assessed using the single factor Kolmogorov–Smirnov (K-S) test. The descriptive statistics, K-S test and variance analysis of SOC were obtained using SPSS 18.0 software. Geostatistical analyses including variogram calculation, independent validation, kriging and mapping were performed using the SGeMS software.

## Results and Discussion

### Soil organic carbon at different profile depths

**Fig 3:**
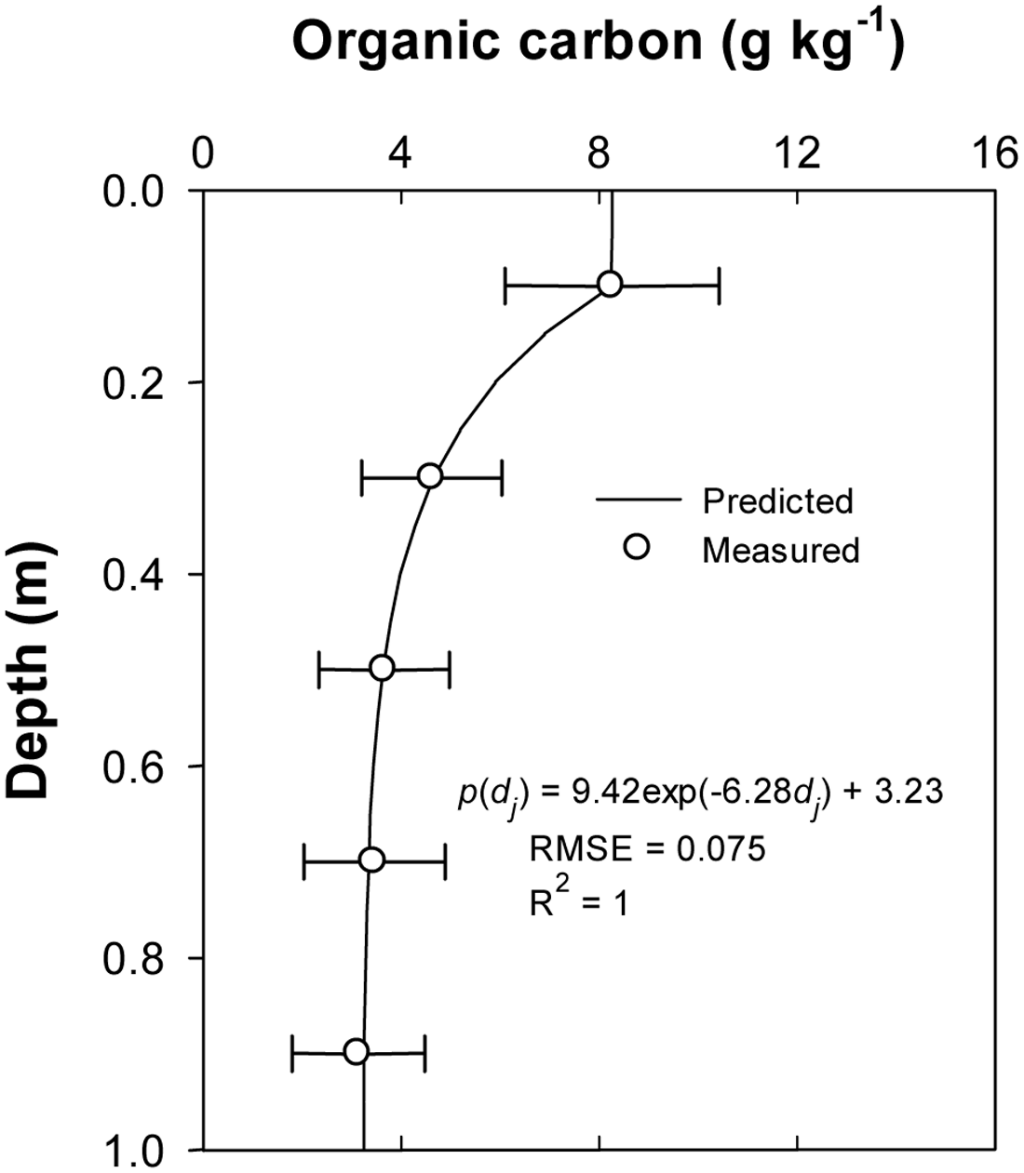
The mean SOC contents at five depths and the corresponding fitting model.

The descriptive statistics and analysis of variance showed that the SOC contents between five different soil depths were significantly different (*P* < 0.05) (Table 1). The mean values of SOC contents gradually decreased from the surface along the soil profile. The mean value of surface (0–20 cm) SOC content was the highest (8.25 g kg^-1^) while the lowest value was measured in the bottom depth (80–100 cm). This variation would be related to the long-term human activities such as tillage, fertilization and crop straw returns that increased the SOC level in the topsoil. The coefficients of variation (CV) of SOC data for all layers ranged from 0.26 to 0.43 and the CV values gradually increased from the surface to the bottom in the soil profile. However, the studies from the Loess Plateau showed that the CV values of SOC data in topsoil were higher than those in subsoil [31, 32]. This difference could be due to the complex textural layers in the alluvial plain in Quzhou County [11]. The results of the K-S test showed that the SOC contents were normally distributed at five soil depths (*P* > 0.05). The analysis of variance showed that the SOC contents were significantly different among soil depths above 0.6 m, while there was no difference in SOC values between soil depths below 0.6 m. The fitting performances (RMSE = 0.075 g kg^-1^, *R* ^2^ = 1) showed that the equation [*p*(*d*_*j*_) = 9.42exp(-6.28*d*_*j*_) + 3.23] could give a reasonable prediction for the means of SOC contents at all five soil depths (Fig 3).

**Table 1:** Statistical characteristics of soil organic carbon in five different depths.

Depth m	Min (g·kg^-1^)	Max (g·kg^-1^)	Mean±SD ^a^ (g·kg^-1^)	CV ^b^	Skewness	Kurtosis	K-S	TD ^c^
							P value	
0–0.2	3.05	17.47	8.25±2.16a	0.26	0.91	2.58	0.09	N
0.2–0.4	1.42	7.89	4.61±1.42b	0.31	0.12	-0.45	0.89	N
0.4–0.6	1.00	6.80	3.65±1.32c	0.36	0.11	-0.43	0.96	N
0.6–0.8	0.92	8.66	3.45±1.43c	0.41	0.92	1.24	0.13	N
0.8–1.0	0.31	7.09	3.14±1.34c	0.43	0.56	0.20	0.48	N

^a^ Standard deviation;

^b^ Coefficient of variation;

^c^ Type of distribution. Different letters in the same column indicate significantly different at *P* < 0.05.

### Prediction and validation of soil organic carbon

**Fig 4:**
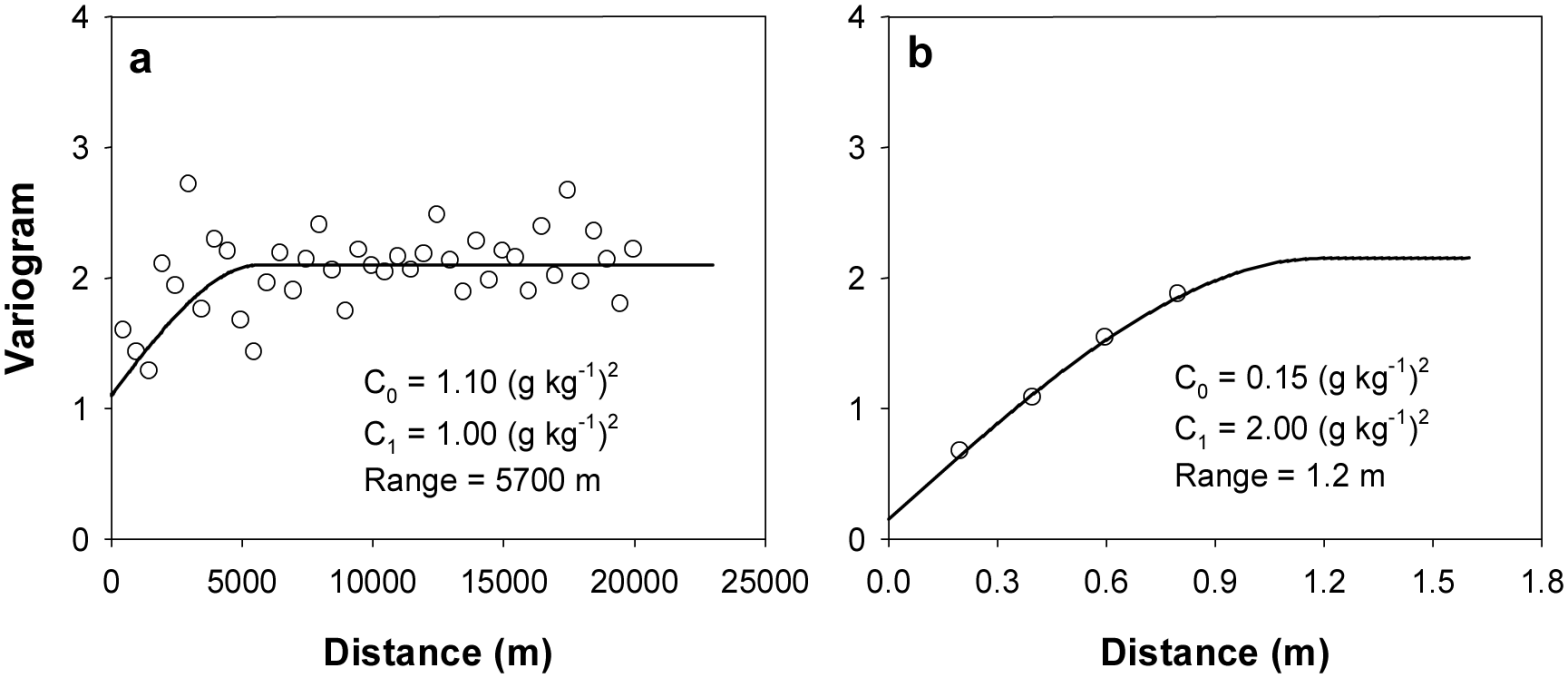
Experimental variograms (circles) and corresponding fitted models (solid lines) for the residual in the two directions (a—horizontal direction, b—vertical direction).

There was no stationary still through calculating the vertical experimental variograms of the SOC data, which meant that it could not be interpolated using the 3D ordinary kriging method. Thus, we firstly subtracted the means for the SOC values at each depth, then fitted the varigorams of the residual *r*(*x*_*αj*_) using spherical model. The omni-directional horizontal (no anisotropy was detected in the horizontal direction) and the vertical variogram models were shown in Fig 4. Then the horizontal and vertical variogram models were combined to construct a 3D anisotropic variogram model, which consisted of an isotropic nugget effect and three nest spherical models (Table 2).

**Table 2:** Parameters of the three-dimensional semivariogram of the residuals.

Model	First nested structure Nugget	Second nested structure Spherical	Third nested structure Spherical	Fourth nested structure Spherical
*C*_0_ (g/g)^2^	0.15	-	-	-
*C*_1_ (g/g)^2^	-	1.00	0.95	0.05
a_horizontal_ (m)	-	5700	0.000001	1000000
a_vertical_ (m)	-	1.2	1.2	1.2

*C*_0_ is nugget value; *C*_1_ is the structural variance; a_horizontal_ and a_vertical_ are the spatial correlation distance in horizontal and vertical directions, respectively.

The interpretation of the 3D variogram model is shown as follows:

1. The first nested structure represents a 3D omnidirectional nugget effect (0.15 g^2^ kg^-2^).
2. A second nested structure represents a 3D anisotropic variogram with an omni-directional sill value of 1 g^2^ kg^-2^ and a directional range. The horizontal range was 5700 m and the vertical range was 1.2 m.
3. The third structure added a nugget effect to the horizontal variogram, due to the extremely short range in the horizontal direction (0.000001 m) and the normal range for the vertical variogram (1.2 m). The combination of the first three structures reproduced the horizontal variogram which is omni-directional in the X–Y plane.
4. The fourth structure increased the sill in the vertical direction without affecting the horizontal direction. This effect was obtained by setting the range in the horizontal direction to a very large value (1,000,000 m), and in the vertical direction to its normal value (1.2 m).

**Fig 5:**
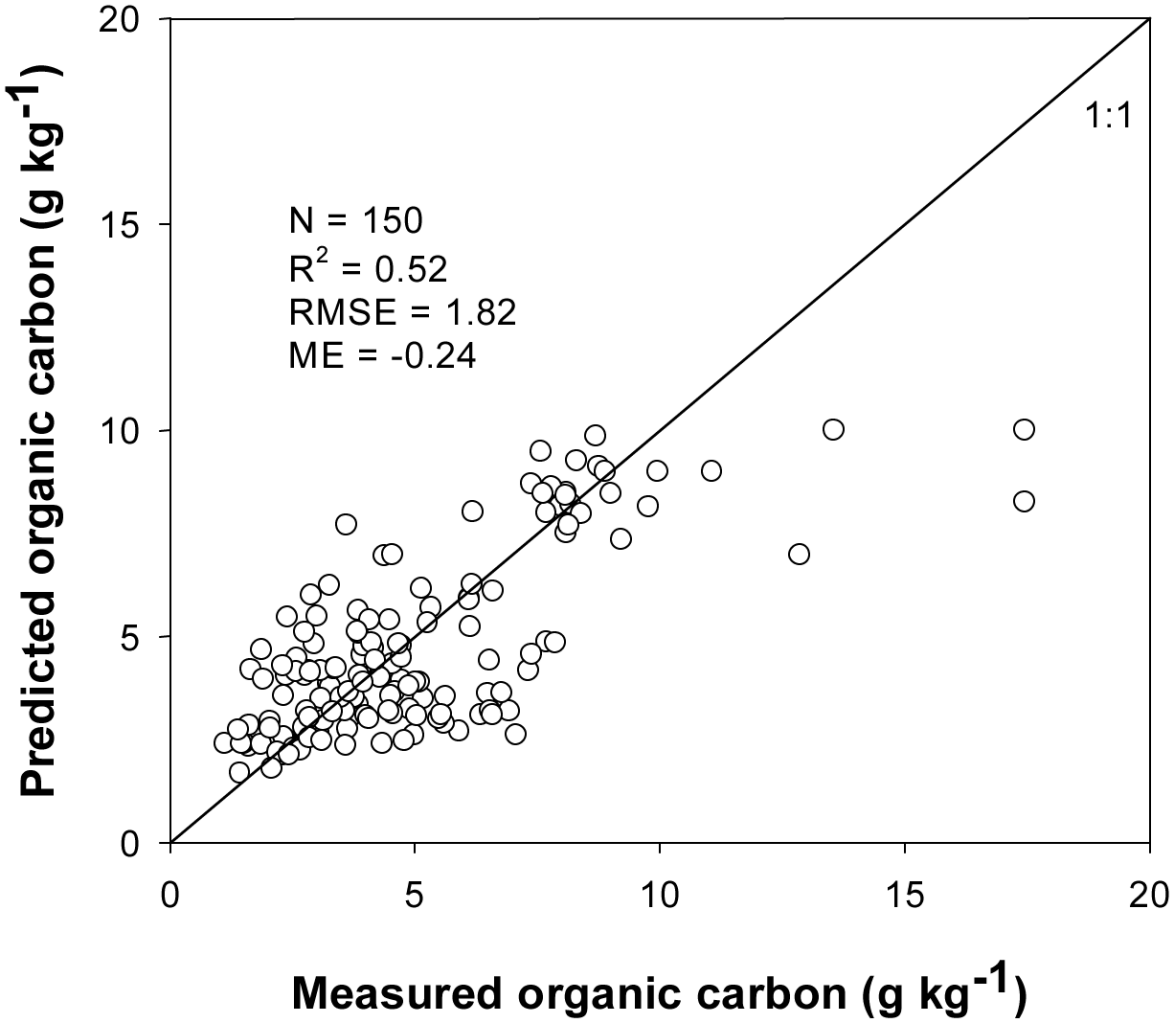
Comparison of the measured SOC content with the predictions using kriging combined with profile depth distribution function (KPDF) interpolation.

Using the 3D variogram model, we obtained the 3D distribution of residual *r*(*x*_*αj*_) after the spatial interpolation. Then the 3D map of SOC was generated by 3D kriging technique combined with profile depth function (KPDF) (Fig 2). The independent validation showed that the measured and the predicted SOC contents were correlated with a significant *R* ^2^ value of 0.52, a small ME value of -0.24 g kg^-1^ and a small RMSE value of 1.82 g kg^-1^ (Fig 5). The Pearson correlation analysis showed that the measured and the predicted SOC had a significant correlation relationship (r = 0.72, *P* < 0.01). Liu et al. [20] obtained the 3D map of SOC in a subtropical hilly landscape by combining spline functions with the RBF neural networks. They reported the MEs of SOC ranged from -0.95 g kg^-1^ to 0.64 g kg^-1^, and the *R* ^2^ values ranged from 0.28 to 0.71. Malone et al. [18] developed a method for mapping the continuous depth functions of SOC storage. They gave the *R* ^2^ value of 0.35 with a validation dataset of 80 profile data. Therefore the prediction accuracy of the KPDF method is comparable to those reported in the literatures, suggesting that it would be useful to produce the 3D map of SOC at regional scale using the KPDF technique. The prediction errors of SOC for few locations were far larger than the other locations when the measured SOC contents were relative higher (Fig 5). Similar phenomenon could also be found in Malone et al. [18]. These phenomena might be due to the smoothing effect of kriging [29].

### 3D distribution of soil organic carbon

**Fig 6:**
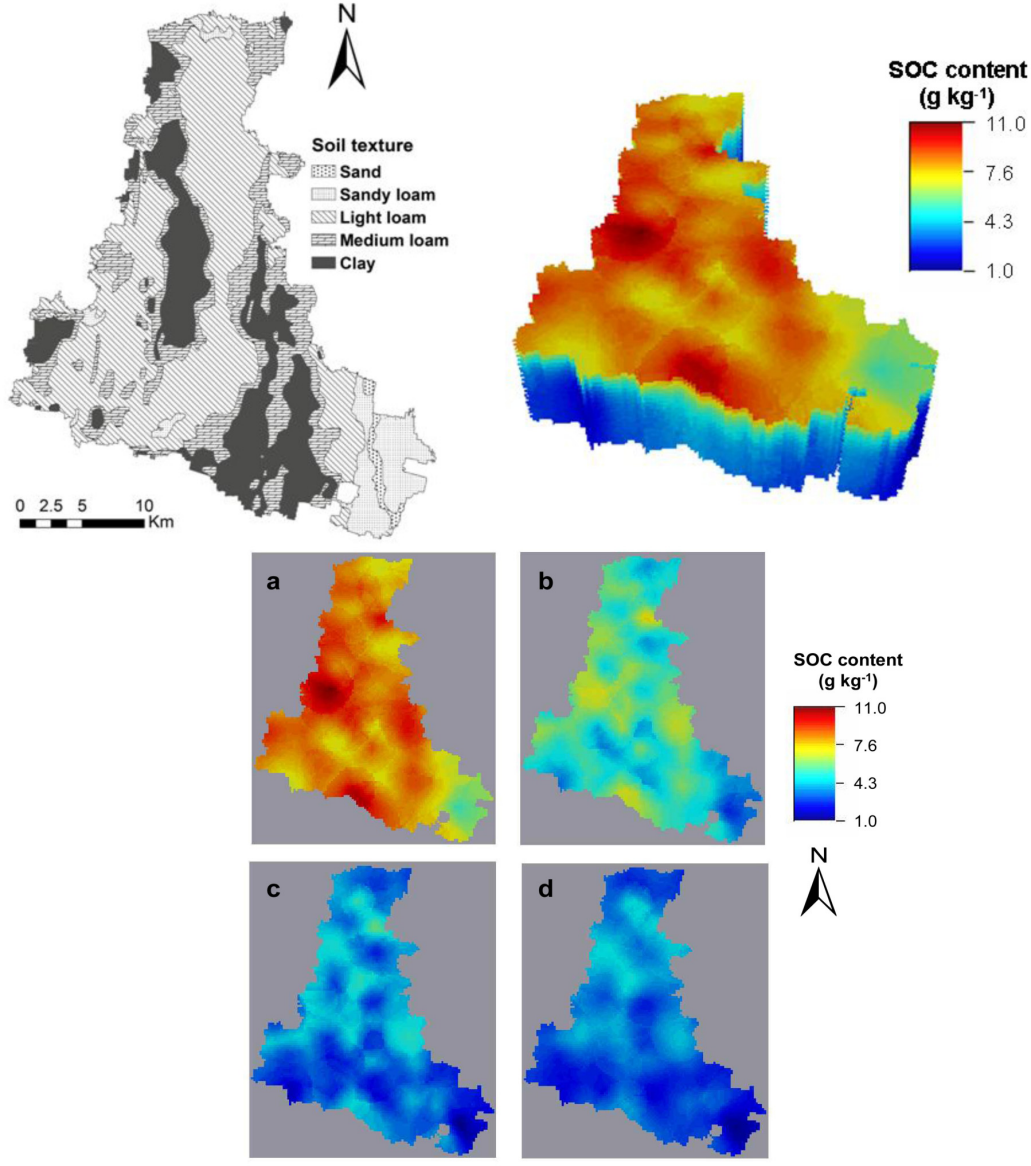
The map of soil texture, three-dimensional distribution map of SOC content in the Quzhou county and the corresponding four cross-section maps of SOC content at 0–0.05 m (a), 0.25–0.30 m (b), 0.55–0.60 m (c), and 0.95–1.00 m (d).

The 3D (grid sizes 300×300×0.05 m) maps showed the corresponding cross-section of predicted SOC contents (Fig 6), where the vertical axis was multiplied by a factor of 10000 to obtain a better visualization of the data interpolation due to the much larger scale in horizontal direction. In the topsoil, the SOC content was high in the mid-west and south areas, and low SOC values lay in the southeast corner. The mid-west area was the agricultural centre of Quzhou County where the vegetables acreage accounted for about 25.2 km^2^, which was the largest in the county [33]. This might be the main reason for higher surface SOC in the mid-west area. The number of livestock in the south area was the largest in the county and organic manure applications for agricultural production could be the main reason for higher surface SOC in this region [33].

The SOC level gradually decreased from surface to bottom (Fig 6), but the tendency of decrease changed at different sites. It was shown that the decrease of the SOC contents in the southeast corner was less than that in the south. The gradual decreasing trends of the SOC contents in different soil layers had certain similarity (Fig 6). The correlation analysis also confirmed that the measured SOC contents among five different depths were significantly positively correlated, especially for SOC in adjacent layers than that in the non-adjacent layers (Table 3). For the investigation of SOC stock at large scale, the number of samples was very large and sampling in deeper layers was not feasible to perform. Based on the correlation of SOC contents between the topsoil and subsoil, it was possible to reduce the number of samples in deeper layers to predict SOC stock.

**Table 3:** Pearson correlation coefficients between SOC content at five different soil layers (0–0.2 m, 0.2–0.4 m, 0.4–0.6 m, 0.6–0.8 m and 0.80–1.0 m).

Variables	Pearson correlation coefficients for SOC at different soil depth profiles				
	0–0.2 m	0.2–0.4 m	0.4–0.6 m	0.6–0.8 m	0.8–1.0 m
0–0.2 m	-	0.66**	0.48**	0.29**	0.32**
0.2–0.4 m		-	0.74**	0.40**	0.32**
0.4–0.6 m			-	0.61**	0.53**
0.6–0.8 m				-	0.80**
0.8–1.0 m					-

** Statistically significant at *P* = 0.01 level (two-tailed).

### The influencing factors of SOC levels

**Fig 7:**
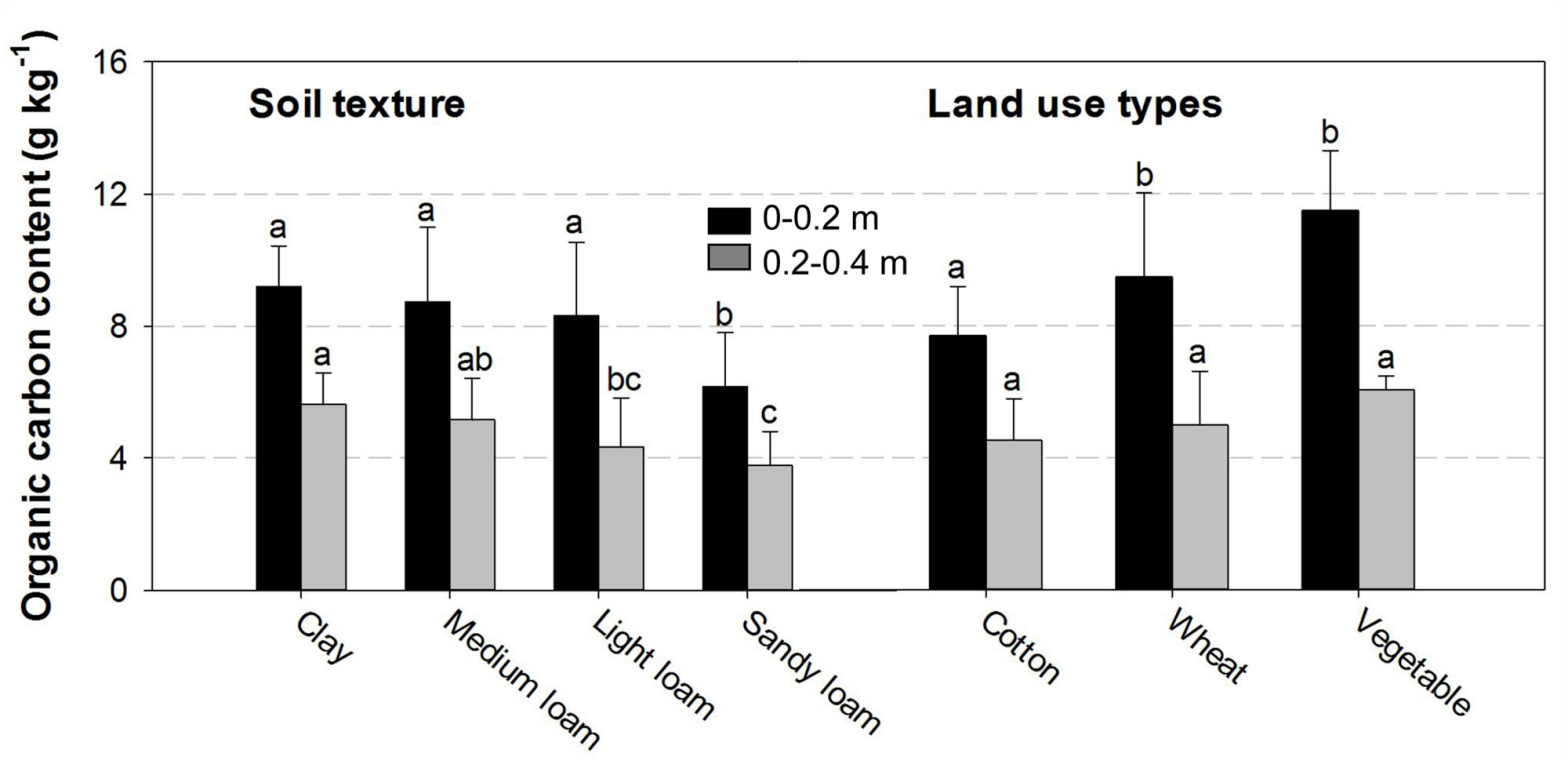
Effects of soil texture and land use types on SOC content. The letters above the bars of a given soil layer denote significant differences between soil texture and land use types for that layer (P < 0.05). The error bars denote the standard errors of the mean.

The influencing factors of SOC levels can be climate, topography, soil texture, land use types, and other micro-scale factors [34–38]. As the county is on a river alluvial plain and the terrain is relatively flat, therefore the effect of topography on spatial patterns of SOC could be ignored, we emphasized on analyzing the effects of soil texture and land use types on SOC levels in the county. Soil texture plays an important role in influencing the amounts and turnover rates of SOC [37, 38]. Compared with climate factors, soil texture had a greater effect on SOC content in Jilin and Liaoning Provinces of China [37]. Zinn et al. [38] found that SOC has a positive linear relationship with silt + clay content in the Brazilian Cerrados. Zhang et al [5] showed that average SOC content for different soil texture was significantly different, and the average SOC content was in the order of loam > light loam > sandy loam > fine sand > sand. In this study, the low SOC level in the southeast corner could be due to the coarser soil texture (sand and sandy loam) (Fig 6). The average SOC content was in the order of clay > medium loam > light loam > sandy loam (Fig 7), meaning that the SOC for soils with fine texture is higher than those soils with coarse texture because fine-grained soils could be easily aggregated, which could protect SOC from decomposition due to adsorption and slow SOC turnover [39]. However, the coarse-grained soils contain less silt and clay that are not conducive to the accumulation of SOC. In addition, high water and fertilizer holding capacity for fine-grained soil can enhance plant biomass, increasing organic carbon in soil [39]. Our results are in agreement with these studies.

In the 0–0.2 m soil layer, no significant difference was found in the SOC levels between the textural classes (clay, medium loam and light loam), and the SOC content in sandy loam was obviously lower than that in clay, medium loam and light loam. However, in the 0.2–0.4 m soil layer, the SOC content of clay was obviously higher than that of light loam, and the SOC contents of light loam and sandy loam had no significant difference. These results showed that soil texture had significant effect on the SOC content in the two layers (0–0.2 m and 0.2–0.4 m), but the influence of soil texture on SOC content in the 0.2–0.4 m layer is stronger than that in the 0–0.2 m soil layer. The reason might be that the impact of human activities in the 0–0.2 m layer was more significant than that in the 0.2–0.4 m soil layer.

The land use types could also be a main influence factor responsible for SOC levels [22–25]. Celik [23] found that the SOC pool of cultivated lands at depth of 0–0.2 m was significantly lower than that in the pasture lands in a southern Mediterranean highland of Turkey. Wiesmeier et al. [24] showed grassland soils had higher SOC stocks than forest and cropland soils in southeast Germany. Hu et al. [6] studied SOC content in 0–0.2 m soil layer under three land use types in Pinggu County of Beijing and found they followed the order of orchard > vegetable> cropland. Zheng et al. [25] also found topsoil SOC content in vegetable was significantly higher than that in cropland in a high-yielding area of northern China.

In this study, there were three main kinds of land use types in the county [24]. Vegetable lands had higher SOC than wheat lands and cotton lands in topsoil (0–0.2 m and 0.2–0.4 m. Fig 7). The highest SOC in the vegetable lands might be due to higher inputs of organic manure, as shown in Hu et al. [6]. The residue would be removed from cotton land after harvesting. However, the residue would be returned to wheat land when harvested. Some studies reported that crop residue returning tended to increase SOC level in soils [35, 40, 41]. The difference of residue handling maybe the reason that wheat land has higher SOC than cotton land in surface soil.

The SOC content for vegetable lands was obviously higher than those of wheat lands and cotton land in the 0–0.2 m soil layer. But there was no significant difference among the three land-use types in the 0.2–0.4 m soil layer (Fig 7). The reason for this SOC variation could be that land use types mainly affected the surface soil SOC level.

We regarded soil texture and land use types as indicators and analyzed their correlation with SOC content, respectively. Soil texture had significant correlation with the SOC in the 0–0.2 m (*r* = 0.30, *P* < 0.01) and 0.2–0.4 m (*r* = 0.39, *P* < 0.01) soil layers. Land use types also had a significant correlation between the SOC level in the 0–0.2 m (*r* = 0.46, *P* < 0.01) soil layer. There was no significant correlation between the SOC in the 0.2–0.4 m soil layer. These results showed that both soil texture and land use types would be the main impact factors controlling the SOC levels at a depth of 0–0.2 m in Quzhou County. Specifically, the influences of land use types were more important than that of soil texture in surface soil, and soil texture could play a more important role in influencing the SOC levels for 0.2–0.4 m layer.

In using profile depth function for representing the vertical distribution of the SOC in whole study area, the effects of soil texture and land use types on SOC were not considered. If we could use a profile depth function for each textural type or land use type, the prediction accuracy of the KPDF might be even improved. It would be useful to verify this assumption in future studies.

## Conclusions

It is novel by combining 3D kriging with the profile depth function of SOC (KPDF) for producing a 3D map of important soil properties such as SOC at a county scale. The exponential equation predictions and an independent validation indicated that the KPDF method could give reliable prediction for the SOC. Soil texture and land use types had significant effects on the spatial distribution of SOC. The influences of land use types were more important than that of soil texture in the surface soil and soil texture could play a more important role in influencing the SOC levels for 0.2–0.4 m layer. The prediction accuracy of the proposed method would be improved by establishing a profile depth function for each textural type or land use type.

## Supporting Information

S1 Datasheet Soil organic carbon (SOC) data in Quzhou County. (XLS)
